# Comprehensive treatment of ear keloid: A case report

**DOI:** 10.1097/MD.0000000000033444

**Published:** 2022-04-07

**Authors:** Meng-Meng Wang, Xiang-Na Cai, Ying-Chang Ji

**Affiliations:** a The First Affiliated Hospital of Shantou University Medical College, Shantou City, Guangdong Province, China.

**Keywords:** comprehensive treatment, ear keloid, radiotherapy

## Abstract

Ear keloid is one of the more common forms of keloid, which may cause pain and itching, and is aesthetically unappealing. Recurrence is common with any monotherapy which prompted a comprehensive, multidimensional approach.

**Patient concerns:**

A 24-year-old female was evaluated in our department on April 6, 2021, due to an “8-year recurrence following a left ear keloid resection.” In July 2013, a left auricle keloid excision was performed in a local hospital. One year following the operation, the scar at the surgical site had proliferated, gradually spreading beyond the original scar borders. Patients worry about recurrence after surgery affecting the appearance of the ear.

**Diagnosis:**

Ear keloid.

**Interventions:**

The patient underwent a 2-stage re-resection of the keloid, followed by postoperative radiotherapy, and triamcinolone acetonide injection around the incision at the time of the second operation. Finally, silicone gel was applied for antiscar treatment.

**Outcomes:**

There has been no postoperative recurrence of ear keloid during the 12-month follow-up.

**Lessons:**

For ear keloids, combination therapy offers an improved approach with an excellent aesthetic appearance and less risk of recurrence than traditional monotherapy.

## 1. Introduction

Keloid scars are common, benign cutaneous complications of wound healing that are often symptomatic and aesthetically unappealing. The pathophysiology is not well understood but involves aberrant collagen deposition that proliferates beyond the original site of trauma. Particularly discouraging to both patient and physician is their propensity for recurrence irrespective of any single treatment modality. Ear keloid is one of the more common forms of keloid, which is readily visible and notoriously recalcitrant to successful resolution. Traditionally, intralesional triamcinolone acetonide corticosteroid (TAC) was administered as monotherapy, but with recurrence rates of 50% at 5 years. The treatment of ear keloids has evolved to utilize surgical resection supplemented by 1 or more adjuvant therapies, such as radiation, steroid injection, laser, cryotherapy, platelet-rich plasma injection, and pressure therapy, to reduce the recurrence rate and achieve better clinical results. This case report presents a combination of staged surgical resection combined with postoperative radiotherapy, TAC injection, and silicone gel application to treat huge ear keloids, with excellent local healing and no recurrence at 12 months of follow-up.

## 2. Case presentation

**Figure 1.**
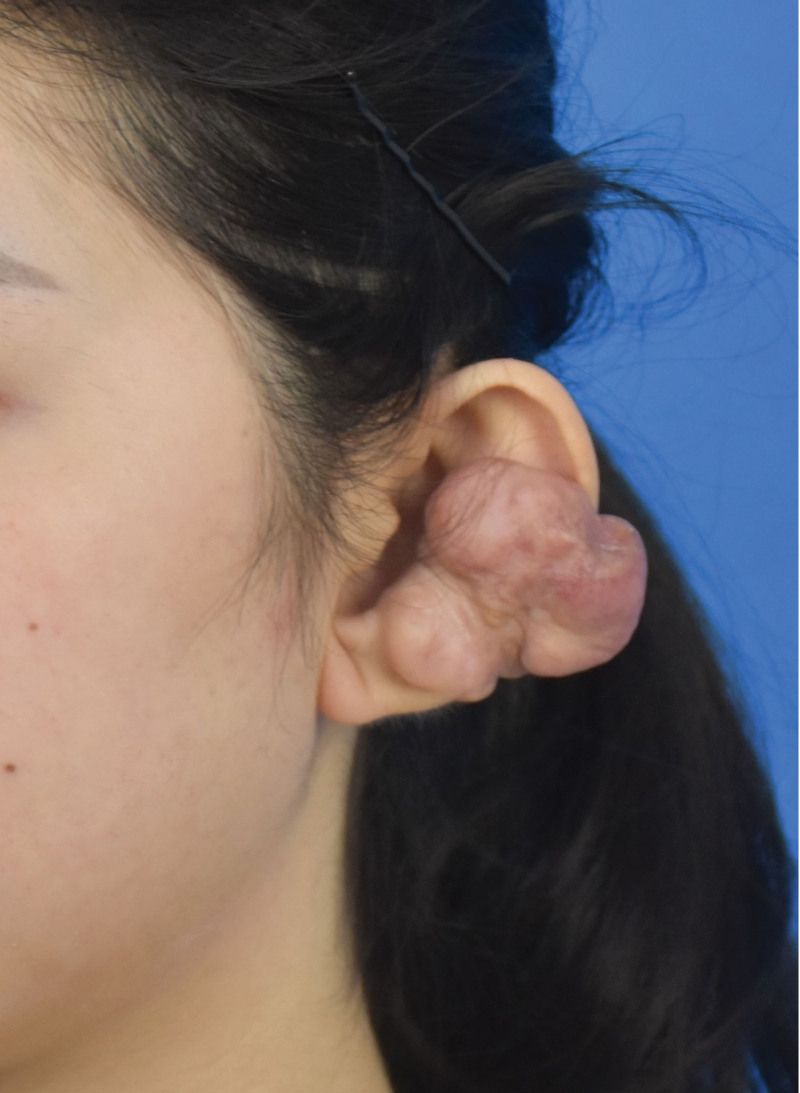
Photo of the patient’s left ear before surgery.

**Figure 2.**
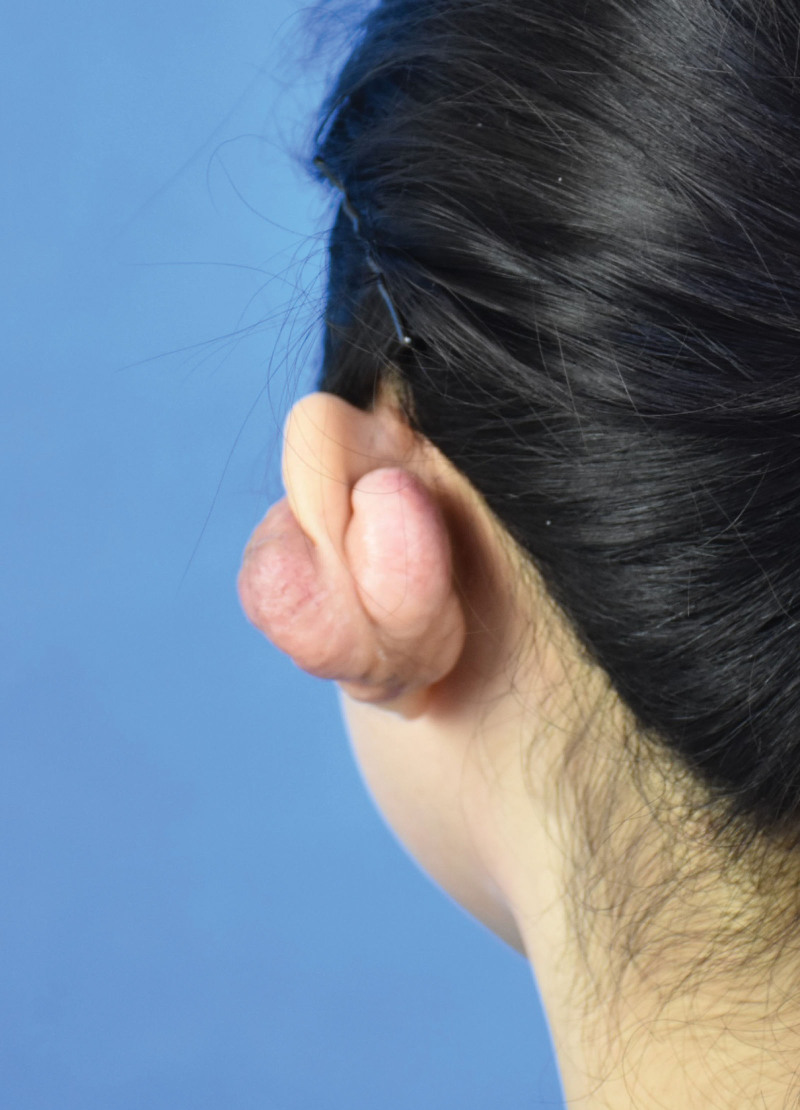
Photo of the patient’s left ear before surgery.

A 24-year-old female was evaluated in our department on April 6, 2021, due to an “8-year recurrence following a left ear keloid resection.” In September 2010, the patient underwent 1 perforation of the left earlobe, followed by a single perforation in the left auricle 6 months later, and several perforations in different sites of the left auricle 4 months later. No infection occurred after perforations. After wearing jewelry for nearly 2 years, scars began to proliferate both anterior and posterior to the 2 ear holes in the left auricle, closing the ear holes. Ultimately, 4 keloids developed in front and behind the auricle, ranging in diameter from 0.3 to 0.7 cm, associated with itching and pain. In July 2013, she underwent left auricle keloid excision in a local hospital, and the pathology results confirmed keloid. There was no further antiscar treatment. One year postoperatively, the surgical scar had proliferated and gradually enlarged on both sides. Approximately 7 years later at our outpatient visit, the keloids had grown to about 5 cm × 4.5 cm (in front of the left auricle) and 4 cm × 2.5 cm (behind the left auricle). The texture was hard, the surface was uneven, and there was capillary hyperplasia, but the skin on the scar surface was intact without ulceration. The photos are shown in Figures 1 and 2.

Surgical management was initiated on April 6, 2021. The surgical resection was confined to the left preauricular keloid only. The patient signed informed consent and the ear was photographed preoperatively. The extent of excision was designed to assure tension-free wound closure. The surgical site was routinely prepared and draped, the area was locally anesthetized, and an incision was made parallel to the external helix, according to the designed incision line. The surface layer was opened, closely separated from the underlying keloid mass, the inner core was excised, and the scar flap was preserved for repair. The scar flap was revised to a suitable size, with uniform thickness, hemostasis was assured, and the wound was sutured, ensuring a tension-free closure. The appearance of the pinna and earlobe was good, and an appropriate pressure dressing was applied. Oral antibiotics were administered for 3 days after surgery. Within 24 hours following the operation, a single anterior field vertical irradiation of a 6 MeV high-energy electron beam was delivered. The irradiation field included 1 cm outside the wound edge, and the surrounding area was protected by a suitable lead mold. The divided dose was 500 cGy/dose, once a day, for 4 consecutive treatments. The sutures were removed 10 days postoperatively.

Four months following the initial keloid resection, the patient returned for resection of the keloid behind the left auricle. Following signed informed consent and photographs, the left posterior auricle keloid was treated in a similar manner as described for the anterior keloid. TAC injection was performed, and the patient has been applying silicone gel antiscar treatment since the second operation.

**Figure 3.**
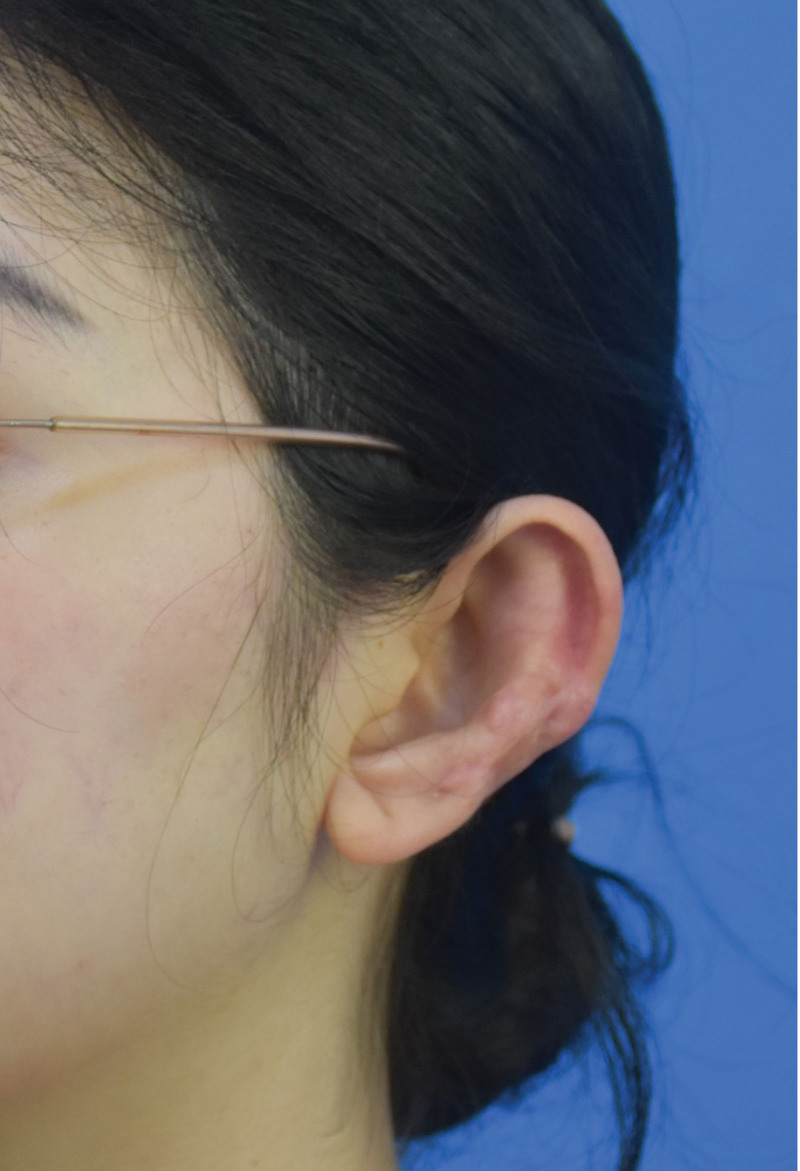
Photo of the patient’s left ear 1 year after surgery.

**Figure 4.**
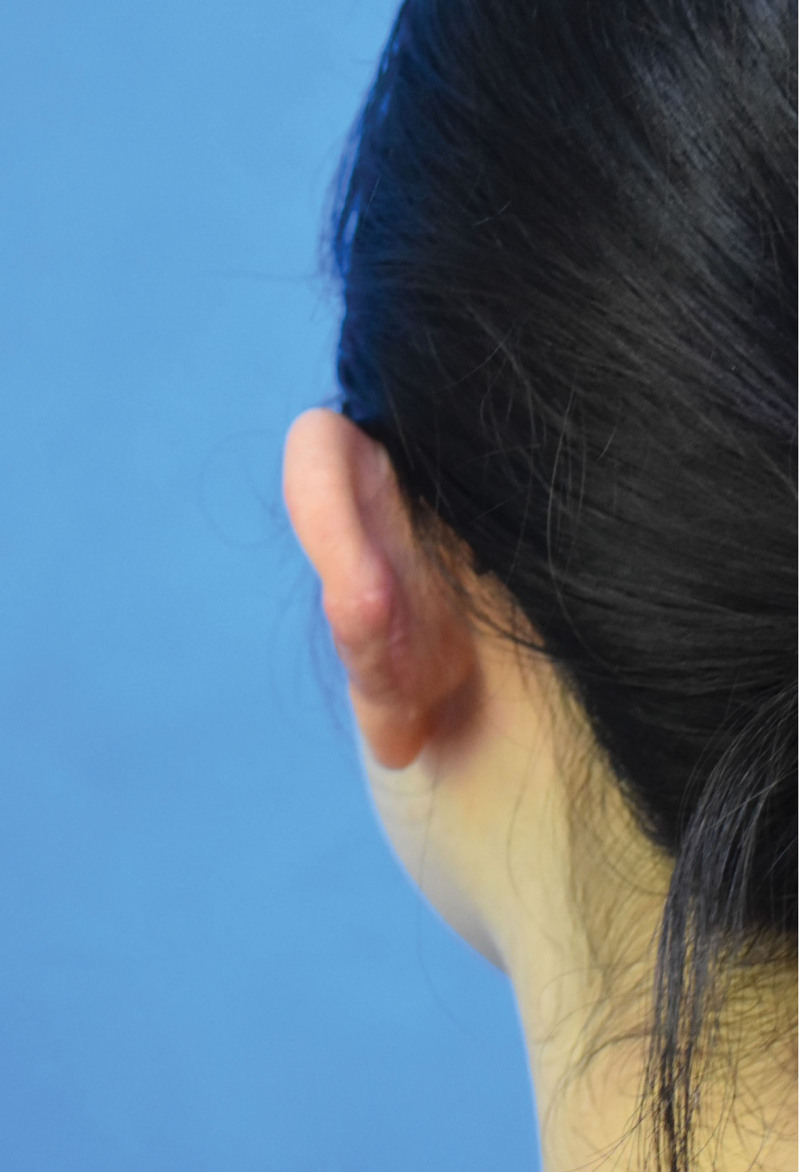
Photo of the patient’s left ear 1 year after surgery.

The patient has been followed up for 12 months after treatment, and the scar has healed well without recurrence. The photos are shown in Figures 3 and 4.

## 3. Discussion

The use of superficial X-ray irradiation to treat keloids was first described by De Bearman and Gourgerot in 1906. Since then, radiation therapy has been widely used as an effective adjuvant therapy following surgical resection,^[1–4]^ with a success rate ranging from 67 to 98%,^[5]^ and a recurrence rate of 22%. Radiation has most often been administered within 24 to 48 hours of surgical resection of the keloid.^[3]^ The mechanism is to inhibit angiogenesis and fibroblast activity, induce fibroblast senescence and apoptosis, and lead to decreased collagen production. Therefore, radiotherapy, as an effective adjuvant therapy, should be implemented as soon as possible after surgery.

If surgical resection is used alone for the management of keloids, rather predictably, the eventual result has been the development of similar or larger keloids,^[6]^ with a recurrence rate as high as 45 to 100%.^[7]^ The clinical course of the presented patient was quite typical of this outcome with relapse developing approximately 1 year following the initial excision, and a recurrent keloid eventually growing significantly larger than the previous one.

Interestingly, the patient’s left posterior auricle keloid significantly softened 1 month after radiotherapy, which reduced the difficulty of the second stage of surgery. It is important to warn patients that immediate radiotherapy following surgical excision of keloids is associated with predictable acute skin side effects of transient erythema, edema, scaling, ulceration, and even necrosis, in almost all patients within the first 7 to 10 days after treatment.^[8]^ This patient was no exception. About 7 days after completing radiotherapy, edema, and ulcers appeared on the skin on the front of the left auricle, which resulted in the suture removal time, nursing requirement, and healing time is significantly prolonged.

There is scarce literature that relates to preoperative radiotherapy for keloids. Levitt and Glilies first reported radiotherapy before and after keloid excision. In 1961, Cosman et al compared the results of combined pre- and postoperative radiotherapy with postoperative radiotherapy alone, with no improvement of the former over the latter.^[9]^ In 2010, the concept of “sandwich therapy” was first reported by Stahl et al.^[10]^ This consisted of surgical resection combined with both pre and postoperative radiotherapy to treat earlobe keloids, which was demonstrated to be effective. From our limited single-case experience, we hypothesized that preoperative radiotherapy might significantly soften keloids, facilitate surgical resection, reduce the predictable complications of postoperative radiotherapy, shorten stitch removal time, nursing requirements, and healing time, and reduce the probability of keloid recurrence. However, we have not found relevant literature evidence as to the efficacy of preoperative radiotherapy for keloids. Long-term follow-up is needed to confirm that keloids so treated have not recurred, and more cases are needed to confirm our hypothesis.

As noted, the patient returned 4 months after the initial resection to treat the keloid behind the left auricle. Because of the short 4-month interval, repeat radiotherapy was not considered safe, and the treatment plan this time was to inject TAC injection around the incision at the time of surgical resection. Since 1961,^[11]^ TAC has been the most widely used corticosteroid for the treatment of keloids.^[12,13]^ Corticosteroids have antiinflammatory and antimitotic properties.^[14]^ Other mechanisms by which corticosteroids reduce keloids include inhibiting fibroblast growth, reducing collagen and glycosaminoglycan synthesis, reducing endothelial cell budding, and enhancing collagen and fibroblast degeneration.^[15,16]^

## 4. Conclusion

There are various treatments for keloids, and emerging treatments are constantly being investigated, but there is still no universally accepted gold standard regimen for treating all keloids. However, combination therapy has been reported to offer the best approach and shows greater efficacy and fewer side effects than monotherapy. Such a comprehensive and relatively intense treatment plan might seem excessive to the patient and even the physician, but the results seem to justify multidimensional approaches.

## Author contributions

**Conceptualization:** Ying-Chang Ji, Xiang-Na Cai.

**Validation:** Ying-Chang Ji.

**Writing – original draft:** Meng-Meng Wang.
